# An overview of the use of plastic-film mulching in China to increase crop yield and water-use efficiency

**DOI:** 10.1093/nsr/nwaa146

**Published:** 2020-07-02

**Authors:** Dongbao Sun, Haigang Li, Enli Wang, Wenqing He, Weiping Hao, Changrong Yan, Yuzhong Li, Xurong Mei, Yanqing Zhang, Zhanxiang Sun, Zhikuan Jia, Huaiping Zhou, Tinglu Fan, Xucheng Zhang, Qin Liu, Fengju Wang, Chaochun Zhang, Jianbo Shen, Qingsuo Wang, Fusuo Zhang

**Affiliations:** Centre for Resources, Environment and Food Security, China Agricultural University, China; Key Laboratory of Dryland Agriculture of Ministry of Education, Institute of Environment and Sustainable Development in Agriculture, Chinese Academy of Agricultural Sciences, China; Centre for Resources, Environment and Food Security, China Agricultural University, China; CSIRO Agriculture Flagship, Australia; Inner Mongolia key laboratory of soil quality and nutrient resources, Key laboratory of grassland resource (IMAU), Ministry of Education, College of Grassland, Resources and Environment, Inner Mongolia Agricultural University, China; CSIRO Agriculture Flagship, Australia; Key Laboratory of Dryland Agriculture of Ministry of Education, Institute of Environment and Sustainable Development in Agriculture, Chinese Academy of Agricultural Sciences, China; Key Laboratory of Dryland Agriculture of Ministry of Education, Institute of Environment and Sustainable Development in Agriculture, Chinese Academy of Agricultural Sciences, China; Key Laboratory of Dryland Agriculture of Ministry of Education, Institute of Environment and Sustainable Development in Agriculture, Chinese Academy of Agricultural Sciences, China; Key Laboratory of Dryland Agriculture of Ministry of Education, Institute of Environment and Sustainable Development in Agriculture, Chinese Academy of Agricultural Sciences, China; Key Laboratory of Dryland Agriculture of Ministry of Education, Institute of Environment and Sustainable Development in Agriculture, Chinese Academy of Agricultural Sciences, China; Chinese Academy of Agricultural Sciences, China; Key Laboratory of Dryland Agriculture of Ministry of Education, Institute of Environment and Sustainable Development in Agriculture, Chinese Academy of Agricultural Sciences, China; Liaoning Academy of Agricultural Sciences, China; College of Agronomy (Academy of Agricultural Sciences), Northwest A & F University, China; Institute of Agricultural Environment and Resource, Shanxi Academy of Agricultural Sciences, China; Institute of Dryland Agricultural Research, Gansu Academy of Agricultural Sciences, China; Institute of Dryland Agricultural Research, Gansu Academy of Agricultural Sciences, China; Key Laboratory of Dryland Agriculture of Ministry of Education, Institute of Environment and Sustainable Development in Agriculture, Chinese Academy of Agricultural Sciences, China; Institute of Crop Science, Chinese Academy of Agricultural Sciences, China; Centre for Resources, Environment and Food Security, China Agricultural University, China; Centre for Resources, Environment and Food Security, China Agricultural University, China; Key Laboratory of Dryland Agriculture of Ministry of Education, Institute of Environment and Sustainable Development in Agriculture, Chinese Academy of Agricultural Sciences, China; Centre for Resources, Environment and Food Security, China Agricultural University, China

Since 1990, crop yields have stagnated in many parts of the world, posing a significant challenge to global food security [1]. As such, innovative solutions are needed to increase the resource-use efficiency of cropping systems to produce more grains per unit area. In China, which is home to one-fifth of the global population, the simple technique of plastic-film mulching (PM) has helped to increase crop productivity by ≤180% in Gansu Province, which can serve as an example to the rest of the world on how simple, cost-effective and innovative solutions can be used to increase food production.

PM was first introduced in the late 1950s and, since the early 1960s, it has been used commercially for vegetable production. However, during the past few decades, this technique has been applied to multiple crop species to attain high yields by conserving soil water and increasing soil temperature. Plastic film has also been developed for use in different types of mulching systems, including flat mulching, ridge mulching, and partial and whole land-surface mulching.

In 2012, ∼13% of China's cropland was mulched, accounting for 60% of the plastic film used for agricultural land mulching worldwide [2]. Many previous studies have focused mainly on improving crop yields and water-use efficiency (WUE) of major crop species, i.e. maize, wheat and potato, by mulching at specific sites or regions [3–6]. However, no comprehensive studies are available that have evaluated the impact of PM on the yield and WUE of all crops across China. Here, we provide a comprehensive analysis of the research undertaken to date and the results of a meta-analysis of the available data assessing the contribution of PM to yield increases for 51 different crop species across China (see Supplementary Data).

## CONTRIBUTION TO CROP YIELD AND WUE

**Figure 1.**
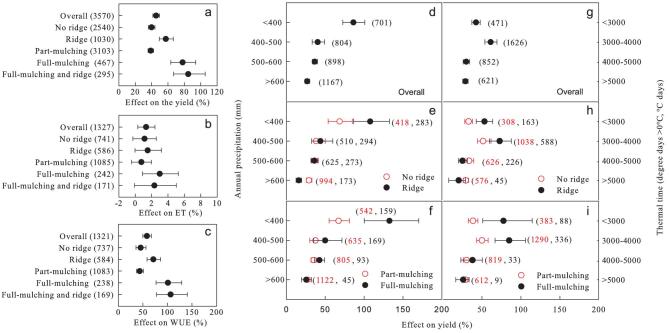
Percent increase in crop yield (a), evapotranspiration (ET) (b) and water-use efficiency (WUE) (c) in response to different types of plastic-film mulching (PM) compared to no mulching, and percent increase in crop yield along precipitation and thermal time gradients (d)–(i). Overall: average of the entire data set; No ridge: part or full mulching without ridges; Ridge: part or full mulching with ridges; Part mulching: only the crop row or land between rows is mulched, with or without ridges; Full mulching: the whole cropland is mulched, with or without ridges; Full mulching and ridge: full mulching combined with ridges. The number of observations for each category is shown in parentheses. The error bars represent 95% confidence intervals.

Nationwide, PM led to a 45.5% increase in crop yield on average (Fig. 1a), with only 5.9% of the cases of PM use having a negative effect (11.3% average decrease in yield) (Supplementary Table 1-1). Part mulching increased crop yields by 39.9% and PM with ridges resulted in an additional 17.4% increase in yield, with a combined increase of 57.4%. In terms of full and partial PM, the yield increase under the former was 77.9% and the yield increase under the latter was 38.9%. The combination of full mulching and ridges resulted in a maximum yield increase of 84.7%. This most effective combination has been frequently adopted in northwestern production areas such as Gansu Province, where both water shortages and cool spring temperatures limit crop growth [7]. In comparison to the other treatments, the part mulching and treatments with no ridges had less of an impact on yield, which was likely due to their minimal impact on water retention and temperature (Supplementary Fig. 1). PM only slightly impacted total water consumption in the field, leading to an average increase in evapotranspiration (ET) of only 1.3% (Fig. 1b). However, no significant difference in ET was detected between the five mulching categories.

The significant yield increase, in combination with the non-significant change in water use in the field, demonstrated increased crop WUE (Fig. 1c). When the all data were pooled, the WUE increased by an average of 58.0%, with a range of increase between 45.3% and 106.4% (Fig. 1c). Among the PM types, the part-mulching and no-ridge treatments had the lowest impact on WUE because they caused the lowest yield improvement.

Water stress is the most limiting factor for crop growth in many parts of China, particularly in the northwestern semi-arid and arid regions. In those regions, ∼80% of the weekly rainfall in the summer, which ranges from 5 to 50 mm [8], can be lost through unproductive evaporation from non-mulched soil or other water-conservation measures. PM is effective at reducing evaporation, while still allowing rainfall water to penetrate into the soil [9]. Ridging provides additional beneficial effects by collecting rainfall and increasing infiltration. Even in relatively humid regions, seasonal droughts can limit crop growth. PM helps to minimize evaporative water loss, divert more water to crop transpiration and reduce crop water stress.

In areas where annual precipitation is <400 mm, PM led to an 86% increase in yield (Fig. 1d). This effect decreased to ∼40% in relatively humid regions whose annual precipitation was ≥400 mm. PM with ridging and full mulching had the greatest effect in the driest areas (<400 mm), leading to yield increases of 108% and 133%, respectively (Fig. 1e and f).

In addition to its water-conservation effects, PM and ridging also help to increase the soil temperature during winter to early spring by reducing heat loss from the soil through long-wave radiation at night. In the northwestern arid and semi-arid regions of China, low air and soil temperatures reduce germination and slow the vegetative growth of major crop species such as wheat [10].

PM can increase the temperature of the topsoil (5-cm depth) by 1.8°C–2.7°C during spring (Supplementary Fig. 1) and even ≤6.8°C in some cases [10]. These increased temperatures result in better germination and subsequently earlier seedling emergence (by 1–3 days for maize, 3 days for wheat and 12 days for potato [7]) and faster crop establishment. Ridges can further increase temperatures by receiving more solar radiation [11]. This effect was generally more pronounced in the cool regions than in the warmer regions (Supplementary Fig. 2a and b). However, yield improvement did not increase from the warm to cool regions (Fig. 1g–i), which may imply that the effect of PM on soil temperature had less of an impact on yield increases than on water. This inconsistency in soil-temperature increase under part mulching and full mulching (Supplementary Fig. 2c) may have been caused by the limited data concerning full mulching and black plastic film used in some studies.

Several studies have shown that PM also enhances the soil-nutrient supply to crops (e.g. soil bioavailable nitrogen (N) and phosphorus) [12] and suppresses weeds [7]. Combined with appropriate tillage practices, PM has a similar effect on herbicides, as PM is able to suppress weed growth by 95% [13]. These effects play a role in increasing crop yield.

## FACTORS INFLUENCING THE EFFECTS OF PM ON CROP YIELD AND WUE

**Figure 2.**
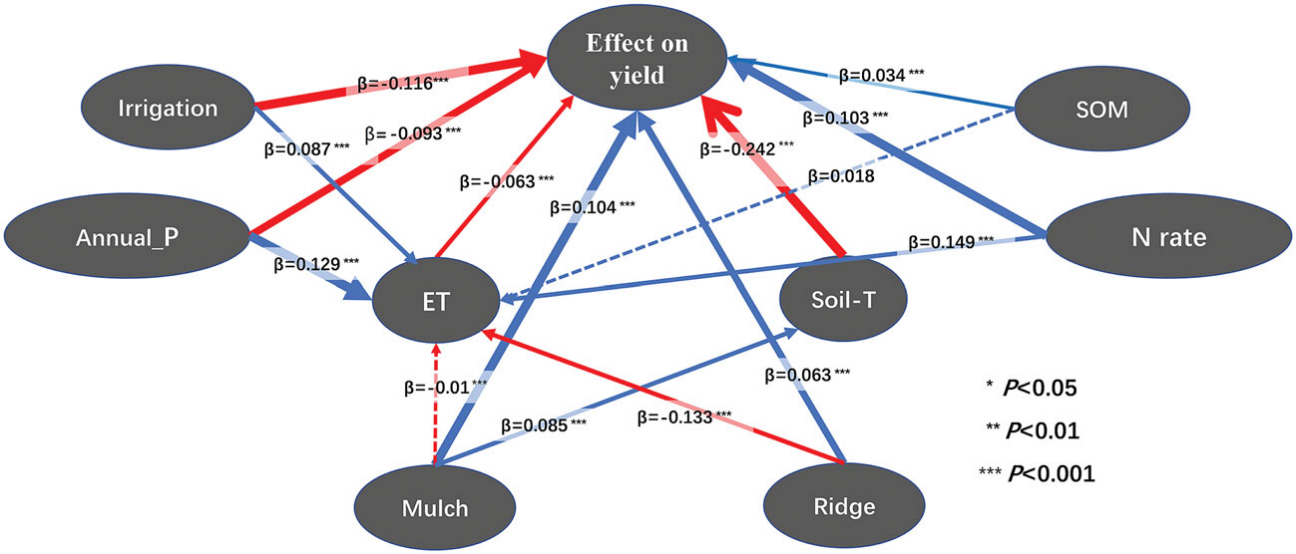
Partial least-squares path models of the factors impacting the effects of mulching on yield. The path coefficients (β values) were computed from regressions and the estimated strength and the directions (red arrow = negative; blue arrow = positive) of relationships between variables were estimated. Effect on yield: effects of mulching on yield, %; Irrigation: rain-fed = 0, irrigated = 1; Annual_P: annual precipitation, mm; ET, mm; Mulch: no mulching = 0, part mulching = 0.5, full mulching = 1; Ridge: no ridge = 0, ridge = 1; Soil-T: mean soil temperature during the crop growth period (0–20 cm), °C; N rate, kg N/ha; SOM: soil organic matter, %.

The effects of PM on yield differed between the various crop species (Supplementary Fig. 4). The results based on current data sets showed that, under PM, the yields of maize and vegetable species increased more than those of other crop species. These results may have been affected by differences in the data sets. Many factors directly and indirectly impact the effects of PM on yield (Fig. 2). Under conditions of increased irrigation (β = −0.116), relatively high annual precipitation (β = −0.093) and relatively high mean soil temperature (soil-T) (β = −0.142), the yield increases were low, with such conditions offsetting the effects of PM. However, PM with ridges increased yield improvements due to minimized water loss and increased infiltration. Sufficient N supply either through fertilizer applications or more N delivery under conditions of relatively high soil organic matter (SOM) contents enhanced the yield improvement in response to PM due to reduced nutrient stress and increased crop growth. Total ET is closely related to total rainfall and increases with irrigation. The decreasing impact of PM with increasing ET may reflect the indirect impact of irrigation and annual precipitation. PM is simple and easy to adopt (manually or at most with a tractor-mounted spooler) yet more efficient and cost-effective than other techniques, especially in regions with an annual precipitation of <400 mm and a thermal time that ranges from 3000 to 4000°Cd (Fig. 1 and Supplementary Fig. 3). PM did not significantly affect ET in areas where the annual rainfall was <400 mm; PM resulted only in a higher WUE.

**Table 1.** The yield benefit from plastic-film mulching in 2012.

Crops	Yield without mulching (kg/ha)^1^	Ratio of yield increase (%)	Yield increase per area of mulching (kg/ha)^1^	Mulched area (ha)^2^	Percentage of mulched area out of the total area (%)^2^	Yield improvement (t)^3^	Equivalent sowing area (ha)^4^
Wheat	3970	32.7 (28.3–38.5)	1298 (1124–1528)	2.4 × 10^3^	0.01	3.1 × 10^3^ (2.7 × 10^3^–3.7 × 10^3^)	0.8 × 10^3^ (0.7 × 10^3^–0.9 × 10^3^)
Maize	7758	58.0 (49.7–67.5)	4531 (3856–5237)	6.2 × 10^6^	17.7	2.8 × 10^7^ (2.4 × 10^7^–3.3 × 10^7^)	3.6 × 10^6^ (3.1 × 10^6^–4.2 × 10^6^)
Rice	6200	28.7 (19.4–38.7)	1779 (1203–2399)	1.0 × 10^6^	3.4	1.8 × 10^6^ (1.2 × 10^6^–2.4 × 10^6^)	2.9 × 10^5^ (2.0 × 10^5^–3.9 × 10^5^)

^1^The arithmetic average of actual crop yields from all studies.

^2^The data of 2012.

^3^Yield improvement = (yield with mulching – yield without mulching) × mulched area.

^4^Equivalent sowing area = yield improvement / yield without mulching; the values in parentheses represent 95% confidence intervals.

## CONTRIBUTION TO NATIONAL FOOD SECURITY

Based on plastic-film-mulched areas in 2012 and the average yield increase under PM, the estimated average increase in total yield was 3.0 × 10^7^ metric tons for the three major grain crop species (wheat, maize and rice) in China. These results are equivalent to the grain production of an additional 3.9 × 10^6^ ha of arable land (Table 1). From 1992 to 2012, the plastic-film-mulched cropland area increased from 4.7 × 10^6^ to 1.8 × 10^7^ ha (Supplementary Fig. 5). This area is likely to further increase, as the use of mulched cropland has been encouraged by the Chinese government because of the significant benefit to crop yields and farmer income, with a net increase in farmer income of 2008–5960 Chinese RMB yuan/ha (Supplementary Table 4).

## NEGATIVE EFFECTS OF PM

Similar to other techniques, the use of PM does have ‘side effects’. Previous applications of PM have led to the accumulation of plastic-film residue in the soil (referred to as ‘white pollution’) (Supplementary Fig. 6), which, in some cases, has resulted in a loss of soil fertility [7] and then a production reduction. Moreover, it can negatively impact several different soil properties, such as the soil water-infiltration rate. High soil temperatures under PM were found to enhance carbon (CO_2_) loss from SOM decomposition and increase N losses (N_2_O, etc.) via nitrification and denitrification [14,15] due to increased soil microbial activity. In addition, long-term plastic mulching was found to increase the salt content of the topsoil and to cause secondary salinization. During the degradation of polyethylene residual mulch film, some environmentally harmful chemical products such as the phthalate ester di-(2-ethylhexyl) phthalate, aldehydes and ketones can form and are then released into the soil. A full life-cycle analysis of PM application has yet to be performed. In addition, the fate of the breakdown of products from PM and their potential impact on soil health and downstream ecosystems remain largely unknown and warrant additional research.

## PROSPECTIVE AND FUTURE WORK

According to both the yield benefits and the environmental risks shown by current PM techniques in China, whether this practice should be encouraged or restricted in certain areas remains unknown. If the negative impacts on the soil and the broader environment from PM can be minimized, then this practice should be extended across China. Fortunately, we are working towards this goal. The Chinese government is promoting and subsidizing the production and application of thicker films (replacing the current 0.005- to 0.007-mm films with 0.010-mm or thicker films), which have improved the recovery ratio from <70% to ∼90% [15], and the development of degradable films, which, like regular films, have positive effects on the yield and water use of crops (Supplementary Fig. 7) but a much higher weight-loss rate (Supplementary Table 1). Owning to lower costs, this approach will allow an increased recovery rate and management of non-degradable films and will minimize waste once degradable films become regularly used.

Improved management practices are also needed to maximize the benefits of PM. For example, optimal PM methods need to be developed for different regions (dry/wet and cool/warm) and purposes (water conservation, temperature modification, weed control, etc.) to optimize the effectiveness and economic viability. As functional films, photo-selective plastic films have begun to be adopted in cash crop production and have shown great potential for improving product quality and controlling diseases. This method has been demonstrating increases in crop yields since 2004 in China. With further improvements, PM could be applied in regions beyond China and, in time, could become a key contributor to advance crop productivity and help secure global food production.

## CONCLUSION

PM improved crop yields and WUE by 45.5% and 58.0%, respectively, across China and contributed 3.0 × 10^7^ metric tons of crop production in 2012. This study can serve as an example to the world on how to increase yields with a simple and cost-effective technique. However, PM has led to the accumulation of plastic-film residue in the soil, which has reduced soil fertility in some cases. This technique needs to be developed further to maximize the benefits and minimize the drawbacks of PM. Thick or degradable films could be options in the future.

## Supplementary Material

nwaa146_Supplemental_File
